# Southern Medical and Surgical Journal

**Published:** 1845-03

**Authors:** 


*Southern Medical and Surgical Journal.* Edited by PAUL F. EVE, M. D., *and* J. P. GARVIN, M. D.

THE above named Journal is published monthly, at Augusta, Ga. Each number contains fifty-six pages. We have received the January, February, March and April numbers of this, the new series. It is handsomely gotten up, and conducted with an ability which reflects the highest credit upon its able editors. Professors Eve and Garvin are connected with the Medical College of Georgia, and their literary and professional abilities are well known and highly appreciated. A Southern Medical Journal is much needed, and we have no doubt that it will be well sustained.

The fourth or April number, contains an able and highly interesting lecture, by professor Eve, on Mesmerism, in which the origin and history of the supposed science are carefully reviewed, and the whole subject discussed with a candor and impartiality that can hardly fail to convince all who read it, that the conclusion to which he comes, is the only rational and correct one that can be arrived at. This is adverse to its pretensions. This paper should be published in pamphlet form, and circulated throughout the length and breadth of the land.—*Balt. Ed.*

